# Operant conditioning of motor cortex neurons reveals neuron-subtype-specific responses in a brain-machine interface task

**DOI:** 10.1038/s41598-020-77090-2

**Published:** 2020-11-17

**Authors:** Martha Gabriela Garcia-Garcia, Cesar Marquez-Chin, Milos R. Popovic

**Affiliations:** 1 Institute of Biomedical Engineering, University of Toronto, Toronto, ON M5S 3G9 Canada; 2 The KITE Research Institute, Toronto Rehabilitation Institute, University Health Network, Toronto, ON M5G 2A2 Canada; 3 CRANIA, University Health Network, Toronto, ON M5T 2S8 Canada

**Keywords:** Extracellular recording, Neuroscience

## Abstract

Operant conditioning is implemented in brain-machine interfaces (BMI) to induce rapid volitional modulation of single neuron activity to control arbitrary mappings with an external actuator. However, intrinsic factors of the volitional controller (i.e. the brain) or the output stage (i.e. individual neurons) might hinder performance of BMIs with more complex mappings between hundreds of neurons and actuators with multiple degrees of freedom. Improved performance might be achieved by studying these intrinsic factors in the context of BMI control. In this study, we investigated how neuron subtypes respond and adapt to a given BMI task. We conditioned single cortical neurons in a BMI task. Recorded neurons were classified into bursting and non-bursting subtypes based on their spike-train autocorrelation. Both neuron subtypes had similar improvement in performance and change in average firing rate. However, in bursting neurons, the activity leading up to a reward increased progressively throughout conditioning, while the response of non-bursting neurons did not change during conditioning. These results highlight the need to characterize neuron-subtype-specific responses in a variety of tasks, which might ultimately inform the design and implementation of BMIs.

## Introduction

Brain-machine interfaces (BMI) exploit the adaptability of the brain to modify cortical circuits for the purpose of controlling neuroprosthetic devices^1–9^. BMIs that explicitly use operant conditioning of neural activity impose an arbitrary task rule between neural activity and external actuators that can be reinforced through rewards. Biofeedback is crucial to exert^10,11^ and maintain^12^ control over the neural activity, down to the single neuron level. Operant conditioning of single neurons has been implemented in humans^13^, non-human primates^14–19^, rats^10,20,21^, and more recently in mice using calcium imaging^12,22–24^. BMIs not only have the potential to replace or augment motor function, but also to be used as tools to study the direct and indirect^12,20,22,25,26^ neural circuits involved in learning as they adapt to new contingencies. Single cortical neurons can be the sole output to a BMI^12,17^, however, recent evidence shows there might be different levels of utility based on the neuron type^27,28^, as well as different strategies to execute a BMI task (e.g. activity up-regulation or down-regulation^24^). Therefore, performance would likely improve with the ability to identify neuron types and subtypes and predict their response prior to their inclusion in a BMI.

Subtypes of cortical neurons can be unequivocally identified in transgenic mice expressing a calcium indicator in specific neuron subtypes using two-photon imaging. In electrical recording studies, different types of putative neurons can be classified based on their extracellular waveform and intrinsic firing dynamics. A bimodal distribution of narrow and wide waveform widths (i.e. trough-to-peak duration of the spike waveform) has been reported in the cortex of rats^29^, non-human primates^30^ and humans^31^. A recent study^27^ found that motor parameters, including kinematics, kinetics and muscle activity, were decoded more accurately and with less neurons using ensembles of narrow waveform neurons, thus the authors proposed waveform width as a predictor of utility in BMIs. However, classification of neuron types based on waveform width alone might be insufficient to distinguish neuron subtypes. For example, wide waveform neurons are known to have two extreme modes of firing behavior: bursting (i.e. spikes are produced in a clustered pattern at time intervals ≤ 5 ms) and non-bursting (i.e. spikes are produced in regular time intervals > 5 ms). Furthermore, numerous studies have investigated the differences in extracellular action potential shape and firing dynamics to distinguish putative pyramidal and interneuron types^29,31–40^, and even putative neuron subtypes (i.e. regular-spiking, fast-spiking, intrinsic bursting, chattering^30^), consistent with those identified in intracellular studies^41^.

In this study, we implemented a BMI in a rat model using operant conditioning of single neuron activity. We conditioned rats to up-regulate the activity of motor cortex neurons using visual feedback. Neurons were classified post-hoc based on two measures of their spike-train autocorrelation histograms into two broad subtypes, which were described as neurons with bursting and non-bursting activity. We investigated the firing rate up-regulation, reward frequency, and the event (i.e. firing rate event that reached or surpassed the reward contingency) amplitude and duration, from which we calculated an event integral as the sum of binned firing rates from the event onset to end. We found that both bursting and non-bursting neurons significantly increased the reward frequency and average firing rates as the experiment progressed, but only bursting neurons did so by gradually incrementing the magnitude of the event integral leading to a reward. Considering these neuron-subtype-specific responses might inform the design of BMIs, as it is a step forward in investigating the inherent capabilities of different neuron subtypes to control a BMI.

## Results

**Figure 1:**
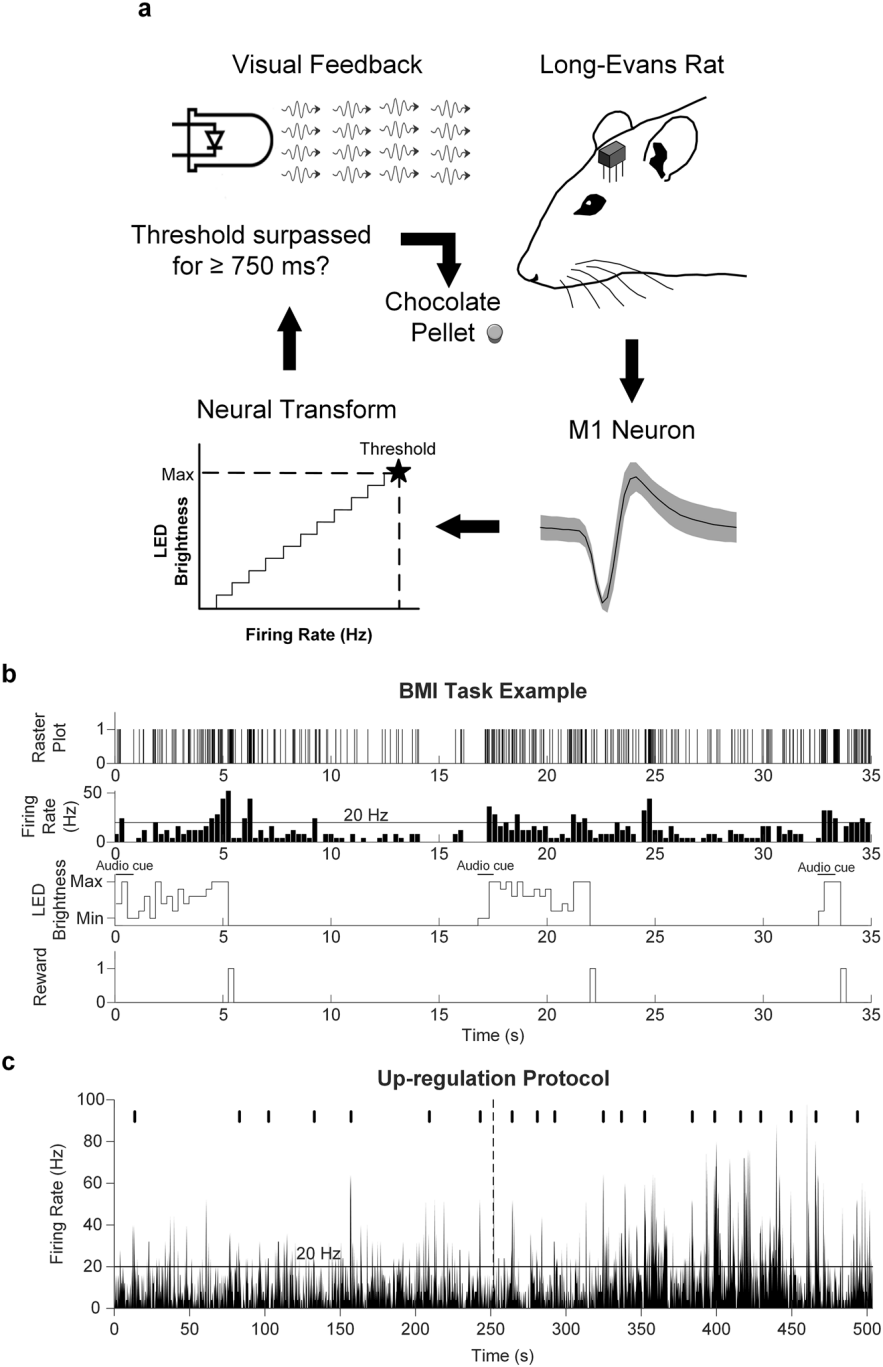
Operant conditioning of single cortical neurons. (**a**) Schematic of the BMI task. (**b**) Real-time transform of the instantaneous firing rates into the brightness of an LED and reward times. Spike times were binned every 250 ms. Rats were rewarded when activity crossed the reward threshold (horizontal line in firing rate histogram) for 3 consecutive bins. (**c**) Example firing rate histogram during an up-regulation protocol (20 trials). Tick marks at the top of the figure denote reward times. The horizontal line marks the high activity threshold. The vertical dotted line denotes half of the protocol. The audio cue is depicted with a horizontal line at trial onset.

We performed electrode implantation in 9 Long-Evans rats, targeting layer V of the forepaw representation in the motor cortex. To condition the single neuron activity, we selected a single unit from the available pool in a given day (see “Methods”). Single neurons were conditioned to progressively increase their average firing rates during the main protocol (i.e. up-regulation), where the instantaneous firing rates were transformed into the brightness of a light-emitting diode (LED). Rats were rewarded when the single neuron firing rate crossed a threshold of 2 standard deviations (s.d.) above pre-BMI baseline activity levels for a minimum of 750 ms (see “Methods”; Fig. 1a,b). The up-regulation protocol consisted of 20 trials (Fig. 1c), which were completed in an average of 10.7 ± 5.1 min (mean ± s.d.). The up-regulation protocol was followed by a second baseline recording (i.e. post-BMI baseline).

### Operant conditioning during the up-regulation protocol resulted in an increase in average firing rates

**Figure 2:**
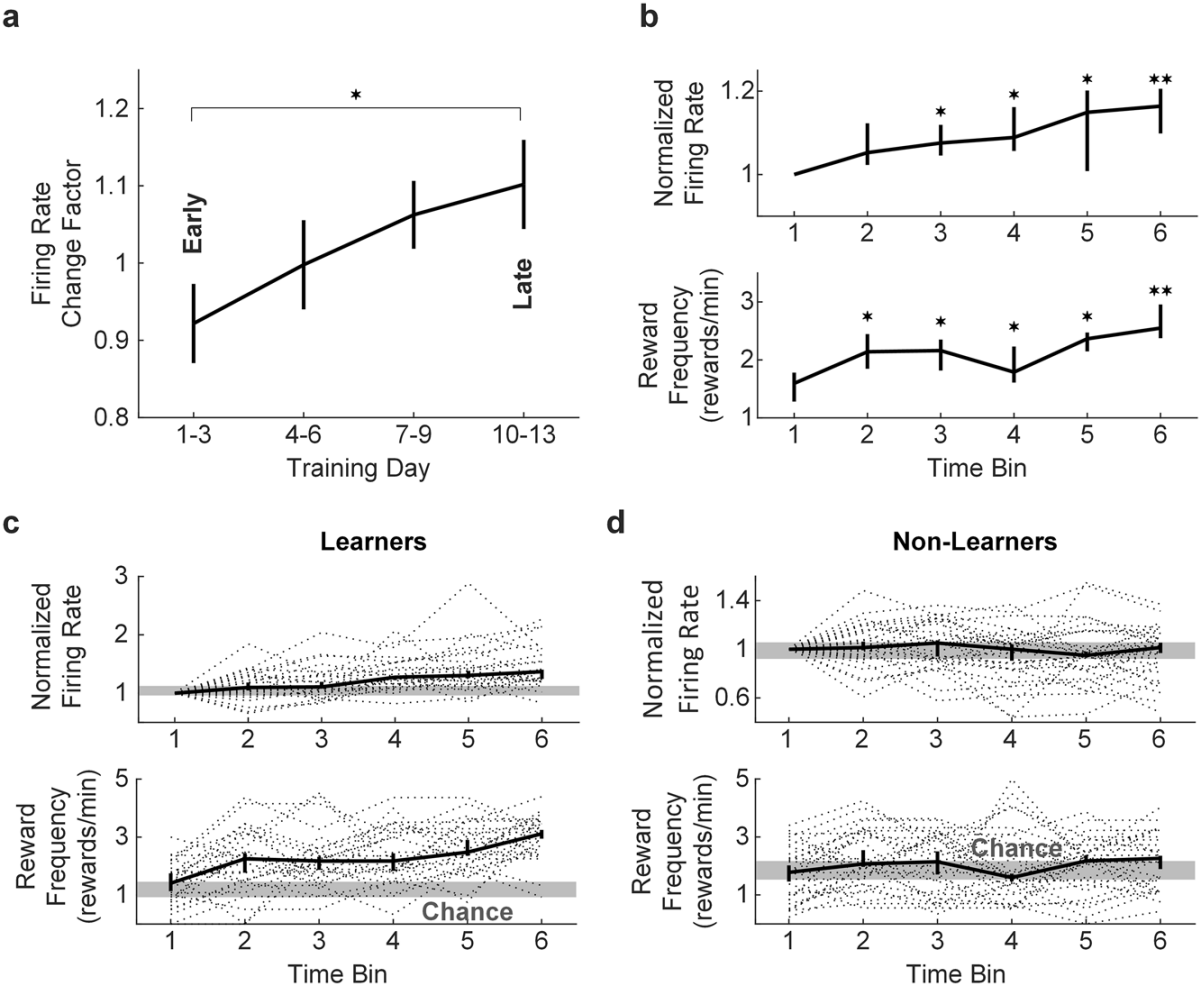
Up-regulation of single neuron firing rates as a result of operant conditioning. (**a**) Learning effect in all 9 rats, depicting an increase in the firing rate change factor during the up-regulation protocol, from early (data from 9 rats) to late training (data from 3–4 rats) (*p < 0.05, unpaired Student’s t test). Data represents mean ± standard error of the mean (s.e.m.). (**b**). Normalized firing rate (*p < 0.01, ** *p* < 1e-04, Wilcoxon’s signed-rank test, Bonferroni corrected) and reward frequency (Wilcoxon’s signed-rank test, *p < 0.01, ** *p* < 1e-05, Bonferroni corrected) for all 57 neurons that underwent operant conditioning, for each of 6 equally-sized time bins. (**c**, **d**) Firing rate and reward frequency for learners and non-learners, for each of 6 equally-sized time bins. The gray bars shows the 40th and 60th percentile range of firing rate and the estimated chance performance, respectively, during pre-BMI baseline. Bold horizontal lines indicate the median and the 40^th^ and 60^th^ percentiles. Gray dotted lines indicate individual neurons.

We conditioned a total of 57 single neurons with a signal-to-noise ratio (SNR) of 3.97 ± 1.31 (mean ± s.d.) from all 9 rats. We ensured that these neurons were single units by looking at absolute refractory period (i.e. 1–2 ms) violations. On average, neurons contained 0.05 ± 0.07% of the total number of spikes in ≤ 1 ms and 0.5 ± 0.6% in ≤ 2 ms. Example of conditioned neuron waveforms are shown in Supplementary Figure S1. On average, rats progressively learned to increase the firing rate of the conditioned neuron during the up-regulation protocol from early (i.e. days 1–3) to late (i.e. days 10–13) training (Fig. 2a; unpaired Student’s t test, *p* = 0.032). The up-regulation protocol of individual neurons was divided into 6 equally-sized time bins for statistical comparison. Neurons had a significant increase in firing rate (Wilcoxon’s signed-rank test, Bonferroni corrected, *p* < 0.01) starting in bin 3, and a significant increase in reward frequency (Wilcoxon’s signed-rank test, Bonferroni corrected, *p* < 0.01) starting in bin 2 (Fig. 2b). These learning-related changes took place in 5.34 ± 2.50 min.

Individual neurons with a significant increase in the firing rate from the first to the second half of the up-regulation protocol (Wilcoxon’s rank-sum test, *p* < 0.05) were classified as learners (Fig. 2c), and non-learners otherwise (Fig. 2d). The presence of significant up-regulation of firing rates was our indicator of successful conditioning and it was found in 27 out 57 (47.4%) neurons. The firing rate during baseline, as well as the chance performance, was not different between learners and non-learners (Wilcoxon’s rank-sum test, *p* = 0.98 and *p* = 0.08, respectively). Also, the SNR of learners (i.e. successfully conditioned neurons) and non-learners (i.e. unsuccessfully conditioned neurons) was not different (unpaired Student’s t-test, *p* = 0.63).

**Figure 3:**
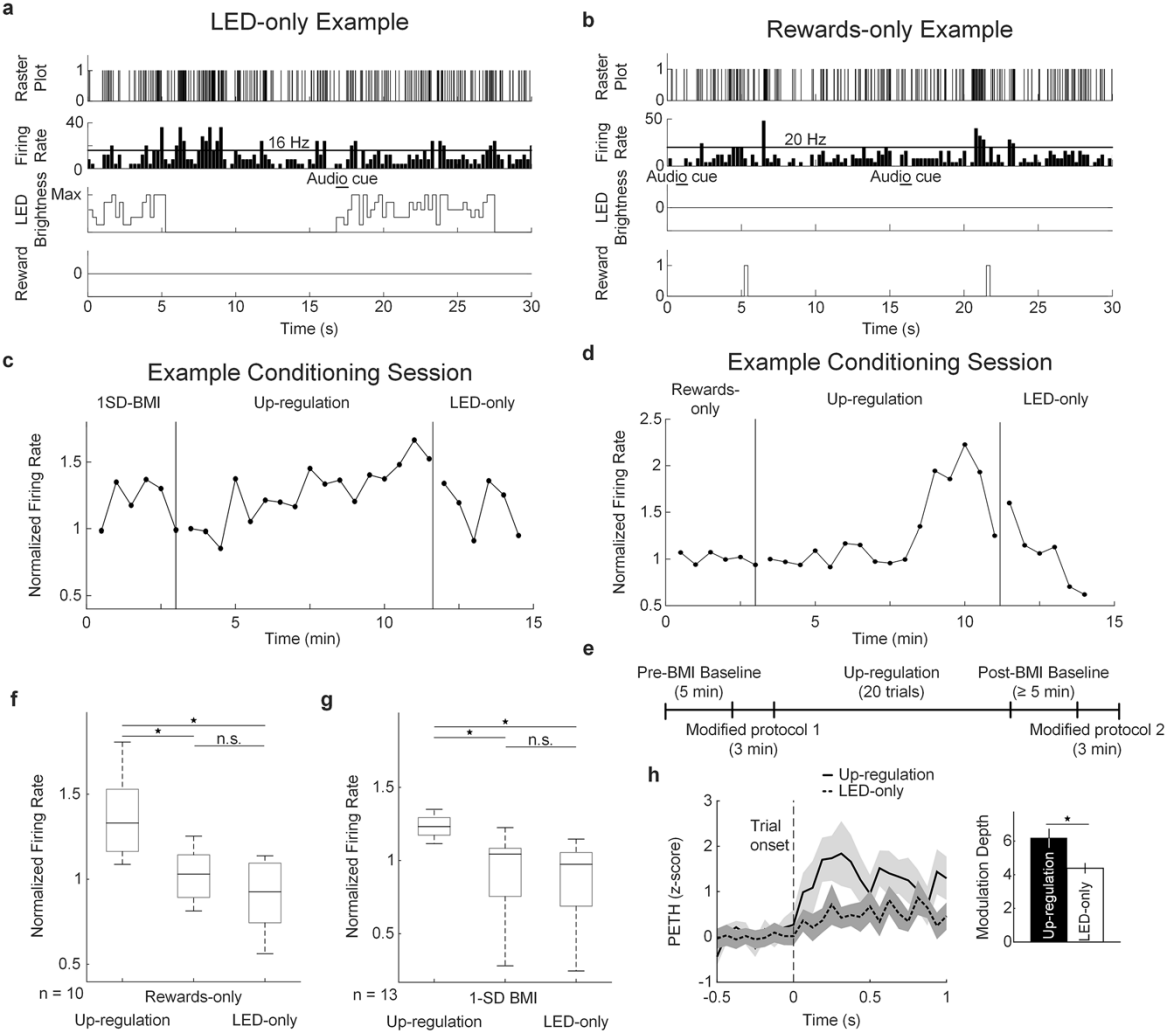
Modified (i.e. control) protocols tested along with the up-regulation protocol. (**a**) Real-time transform of firing rates during the LED-only protocol. (**b**) Real-time transform of firing rates during the rewards-only protocol. (**c**) Thirty-second firing rate averages during example conditioning session where 1-SD BMI and LED-only protocols were tested. Pre-BMI and post-BMI baseline firing rates are not shown. (**d**) Thirty-second firing rate averages during example conditioning session where rewards-only and LED-only protocols were tested. Pre- and post-BMI baseline firing rates are not shown. (**e**) Experimental timeline. Modified protocols were randomized within and across sessions (see “Methods”). (**f**) Firing rates during the rewards-only and LED-only protocols were significantly lower than the last 3 min of the up-regulation protocol (Wilcoxon’s signed-rank test, Bonferroni corrected). Boxplots depict the median and quartiles. (**g**) Firing rates during the 1 SD-BMI and LED-only protocols were significantly lower than the last 3 min of the up-regulation protocol (*p < 0.017, n.s. not significant, Wilcoxon’s signed-rank test, Bonferroni corrected). Boxplots depict the median and quartiles. (**h**) Average *z*-score peri-event time histogram (PETH) aligned to trial onset, from -0.5 to 1 s during the up-regulation and LED-only protocols and corresponding modulation depth (*p < 0.05, paired Student’s t-test). The gray contours depict ± s.e.m. Bars are mean ± s.e.m.

To validate that the BMI was indeed under volitional control, we tested the effect of visual feedback and rewards on the neuron’s firing rate in 3 different modified (i.e. control) protocols: (1) LED-only (i.e. no rewards; Fig. 3a), (2) rewards-only (i.e. no LED feedback; Fig. 3b), and (3) 1 SD-BMI (i.e. rewards and LED feedback with a reward threshold of 1 s.d. above baseline firing rate). Two protocols were tested in a given conditioning session, one before and one after the up-regulation protocol (Figs. 3c–e; see “Methods” for details on modified protocol order randomization). We found that the up-regulation protocol of learners had significantly higher firing rates compared to the first paired set of modified protocols (Fig. 3f): rewards-only (Wilcoxon’s signed-rank test, Bonferroni corrected, *p* = 0.002) and LED-only (*p* = 0.0098). A similar effect was found in the second set of paired modified protocols (Fig. 3g), where the up-regulation protocol of learners had higher firing rates compared to: 1-SD BMI (*p* = 0.0002) and LED-only (*p* = 0.0004). No significant difference was found between modified protocols (*p* = 0.70, *p* = 0.59, respectively). These results suggest that successful up-regulation was a consequence of learning the BMI task, rather than a simple response to light feedback or reward expectation. Also, up-regulation only occurred when a higher, more challenging reward threshold was introduced in the contingency.

In addition, learners had slight modulation to the audio cue (i.e. beep) at trial onset, but only during the up-regulation protocol. This modulation was not present during the modified protocol LED-only, where rewards were not given to the rat upon reaching the contingency (Fig. 3h). We found that the modulation depth at trial onset was significantly different between the LED-only and up-regulation protocols (paired Student’s t-test, *p* = 0.025). In learners, a histogram of the times to reach or surpass the reward contingency shows that the percentage of trials completed in the minimum time (i.e. 750 ms) was 2.2% in the first half of the up-regulation protocol, while in the second half it increased to 4.6% (Supplementary Figure S2), demonstrating that modulation at trial onset was not sufficiently robust to dispense a reward. Although, this slight increase at onset in trials involving rewards suggests that modulation was volitional.

### Direct neurons were strongly modulated and led activation compared to indirect neurons around the reward contingency

**Figure 4:**
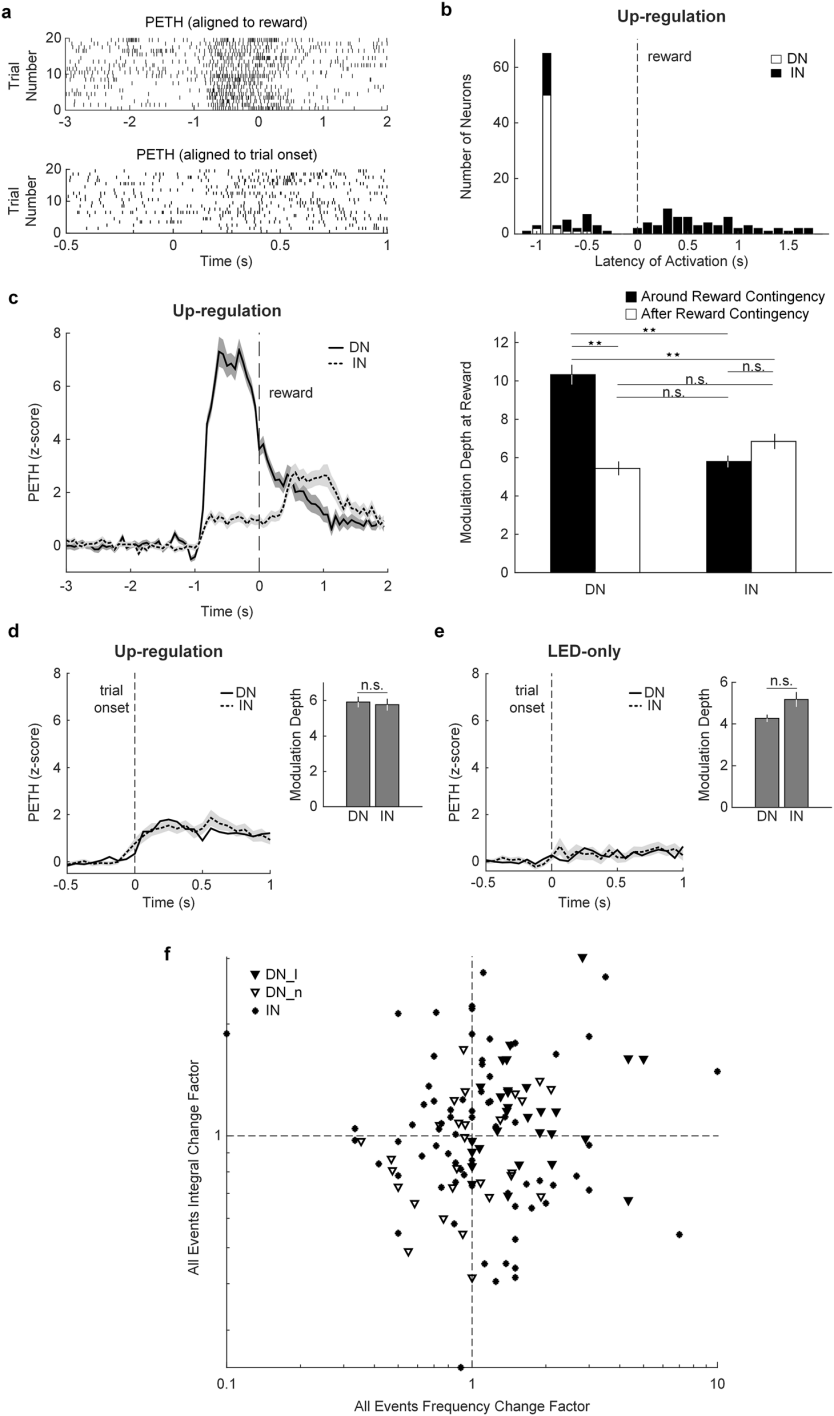
Activity of direct and indirect neurons around and after reward. (**a**) Example peri-event time histogram (PETH) for a direct neuron (DN) during the up-regulation protocol aligned to reward dispensing (top) and trial onset (bottom). (**b**) Latency of activation of all DNs and INs. (**c**) Average *z*-score PETH trace for sessions where INs were detected along with the DNs. The mean traces are marked with bold and dashed lines, while the gray contours denote ± s.e.m. for DNs and INs, respectively. The corresponding modulation depth for DNs and INs around and after the reward contingency is shown in the right. The modulation depth was significant around the reward contingency for DNs. Also, DNs were significantly modulated around the reward contingency compared to INs. Bars are mean ± s.e.m. Two-way ANOVA and Tukey’s HSD post-hoc test for multiple comparisons, **p < 1e-07, n.s. not significant. (**d**, **e**) Average *z*-score trial onset-aligned PETH traces for DNs and INs during up-regulation and LED-only protocols. The modulation depth is shown at the right of each trace (n.s. not significant, unpaired Student’s t-test). Bars are mean ± s.e.m. (**f**) All event (i.e. firing rate event that reached or surpassed the contingency) integral and frequency change factors for learner (DN_l) and non-learner (DN_n) DNs (downward facing arrow symbols) and INs (star symbols). The dashed lines on both axes depict a factor change = 1 (i.e. gain > 1). Axes are shown in logarithmic scale.

In all conditioned neurons (i.e. learners and non-learners), we investigated if activation was exclusive to the neuron directly under conditioning (i.e. direct neuron; DN) or if the response was generalized to the local circuit (i.e. indirect neurons; INs), which might be the result of stereotyped forepaw movements that drive neural activity at the recording site. First, we computed a peri-event time histogram (PETH) for DNs and INs aligned to reward (Fig. 4a). We found that the activation latency of DNs often preceded that of INs recorded in the array (Fig. 4b). Out of a total of 512 neurons recorded from the arrays of all 9 rats (SNR of 3.37 ± 1.06), 94 (18.4%) INs were found to be active either before or after the reward contingency was met. Out of the 94 INs, 33 (35.1%) had activation latencies before the reward contingency was met, while the majority of INs (61; 64.9%) had activation latencies after the reward contingency was met. The average *z*-score PETH traces for all DNs and INs are shown in Fig. 4c.

Next, we looked into the modulation depth of DNs and INs (Fig. 4c) around the reward contingency (i.e. − 1 to 0.5 s), and after the reward contingency (i.e. 0.5–2 s) given that the majority of INs had latencies of activation in this time window. We found that the modulation depth of DNs was significantly greater than the modulation depth of INs around the reward contingency (two-way ANOVA, *F*_1,298_ = 14.84, *p* = 0.0001; Tukey’s HSD post hoc test for multiple comparisons, *p* = 3.77e-09). Also, a significant increase in the modulation depth of DNs was found around the reward contingency (two-way ANOVA, *F*_1,298_ = 22.43, *p* = 3.37e−06; Tukey’s HSD post hoc test for multiple comparisons, *p* = 3.77e−09), indicating that DNs were strongly modulated and this response was specific to reward dispensing, as activation quickly died down after the reward contingency was met. On the other hand, changes in the modulation depth of INs were not significant after reward contingency (Tukey’s HSD post hoc test for multiple comparisons, *p* = 0.068). DN activation was not different than IN activation after the reward contingency (Tukey’s HSD post hoc test for multiple comparisons, *p* = 0.15).

We also investigated DN and IN modulation at trial onset (Fig. 4a). The average *z*-score traces of the trial onset-aligned PETH of DNs and INs show that both neuron types were modulated to a similar extent at trial onset during the up-regulation protocol (Fig. 4d). As expected, no difference in modulation depth was found between DNs and INs at trial onset during the up-regulation protocol (unpaired Student’s t-test, *p* = 0.76). Similar to DNs, IN modulation was not present during the LED-only protocol, in which rewards were not given upon reaching the reward contingency (Fig. 4e). No difference was found in the modulation depth of DNs and INs at trial onset during LED-only (unpaired Student’s t-test, *p* = 0.067).

Finally, we simulated the event (i.e. firing rate event that reached or surpassed the contingency) frequency and integral (i.e. sum of binned firing rates from event onset to end) for INs, using the same criteria applied for DNs (i.e. 2 s.d. above baseline firing rate for ≥ 750 ms). While IN events were unrewarded, a few INs underwent similar changes observed in DNs during the up-regulation protocol. On the other hand, DNs produced events spontaneously while the rats were retrieving the reward during the 10 s in between trials, when the LED feedback was turned off (see “Methods” for details on trial structure). These unrewarded events became more frequent as the up-regulation protocol progressed and were only observed in between trials because a new trial would not start until the firing rate went back to baseline. Therefore, the only event found during a given trial was the rewarded event.

We quantified the change factor in the frequency and integral of all events (i.e. rewarded and unrewarded) for DNs and INs from the first to the second half of the up-regulation protocol (Fig. 4f). We found that the proportion of DNs (i.e. 27 out of 57) with frequency and integral change factors > 1 (i.e. gain) was significantly larger (χ^2^ (1, 134) = 8.45, *p* = 0.0036) than that of INs (i.e. 18 out of 77). Moreover, the proportion of DNs with significant up-regulation (i.e. learners) with change factors > 1 (i.e. 19 out of 27) was significantly larger than that of unsuccessfully conditioned DNs (i.e. non-learners) and INs (χ^2^ (2, 134) = 22.85, *p* = 1.09e-05).

### Neurons were classified into bursting and non-bursting types using autocorrelation-based measures and *k*-means clustering

Next, we investigated whether the response of bursting and non-bursting neurons differed as a result of conditioning. We measured the response as the changes in average firing rates, reward frequency, the event frequency and the event integral during the up-regulation protocol.

**Figure 5:**
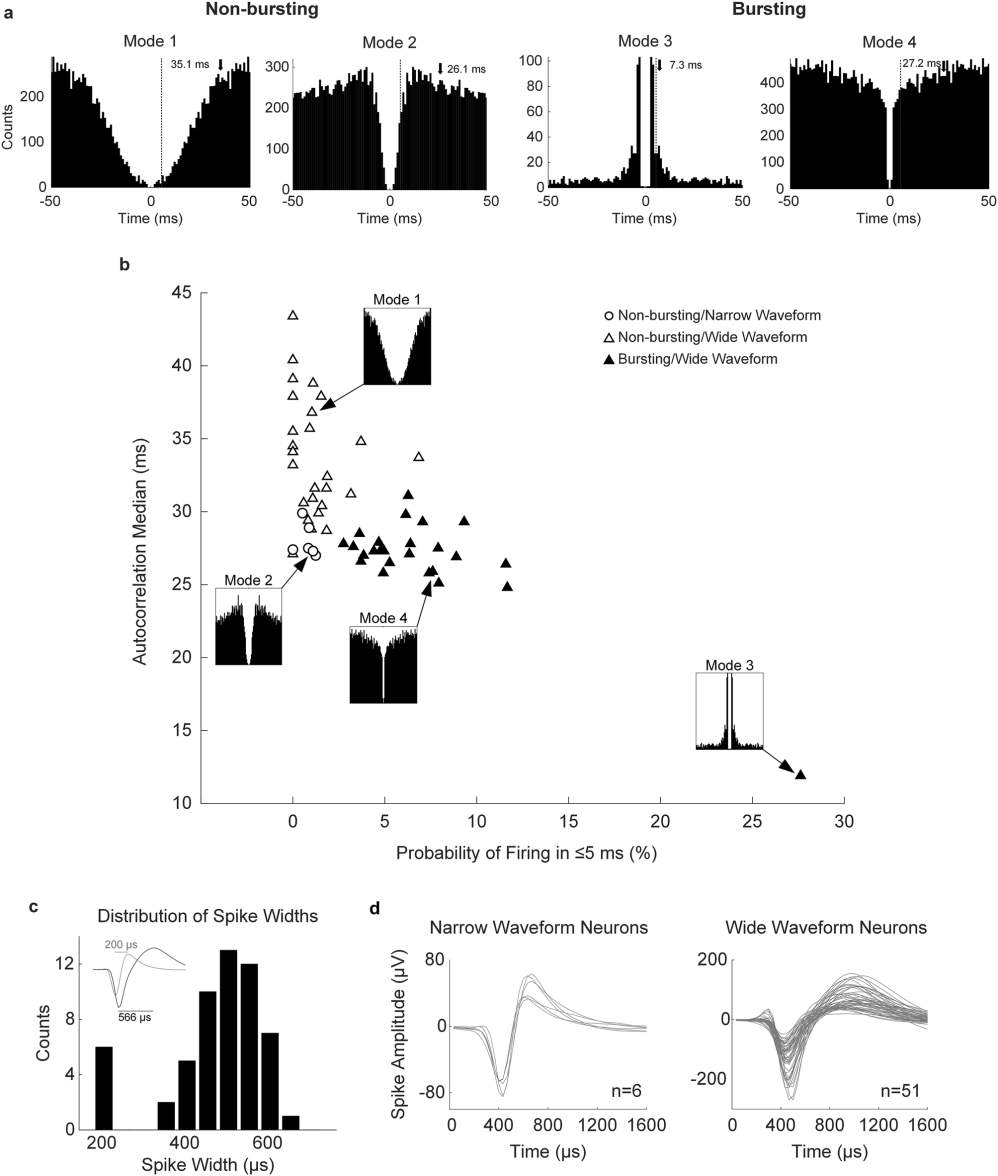
Neuron classification based on spike-train autocorrelation histogram measures and the trough-to-peak waveform width. (**a**) Four predominant modes of firing were identified from the spike-train autocorrelation histograms of all 57 neurons in the dataset. All available spikes were used to construct the autocorrelation histograms for visual inspection. Autocorrelation histograms have a resolution of 1 ms. The autocorrelation median was calculated with 0.1 ms resolution and is marked with a downward-facing arrow. The vertical dashed lines denote the 5 ms threshold. (**b**) *k*-means clustering showing bursting and non-bursting neurons, with the probability of firing in ≤ 5 ms in the x-axis and the autocorrelation median in the y-axis. Only the first 350 spikes from pre-BMI baseline were used to compute the autocorrelation-based values to facilitate between-unit comparisons. The predominant modes of firing are shown in the location where they were found in the *k*-means plane. Classification based on waveform width is shown with different symbols: triangles for wide waveform neurons, and circles for narrow waveform neurons. (**c**) Distribution of waveform widths with a clear cut-off between narrow and wide waveform neurons. Inset shows the difference in trough-to-peak width between narrow and wide waveform neurons. (**d**) Traces of narrow waveform and wide waveform neurons.

First, to classify neurons, we computed the spike-train autocorrelation histogram from which we extracted two measures: the autocorrelation median (i.e. positive time lag where half the total histogram counts occur), and the probability of firing in ≤ 5 ms (i.e. percentage of histogram counts in ≤ 5 ms from the total 50 ms autocorrelation histogram counts). An intracellular recording study^41^ demonstrated that regular-spiking neurons, which do not produce bursts, have long refractory periods (i.e. 5 ms) compared to other neuron subtypes. Another intracellular recording study reported that in the cortex only excitatory pyramidal neurons produce bursts^42^. In addition, an extracellular recording study used the activity in ≤ 5 ms to split non-bursting from bursting neurons^30^. These measures (i.e. autocorrelation median and probability of firing in ≤ 5 ms) were chosen based on these criteria, as well as on visual inspection of the four main modes of neuron firing identified from the dataset (Fig. 5a; see “Methods” for details regarding the modes of firing identified from the spike-train autocorrelation). Two modes described non-bursting neurons (i.e. Modes 1 and 2), while the remaining two described neurons with bursting propensity (i.e. Modes 3 and 4). Spike trains from each mode of neuron firing are shown in Supplementary Figure S3. Next, neurons were clustered into non-bursting and bursting types with a *k*-means algorithm (Fig. 5b), based on the autocorrelation-based measures described above. Bursting neurons (n = 24) had a probability of firing in ≤ 5 ms of 7.32 ± 4.90% and autocorrelation median of 26.69 ± 3.48 ms. Non-bursting neurons (n = 33) had a probability of firing in ≤ 5 ms of 1.18 ± 1.39% and autocorrelation median of 32.55 ± 4.37 ms. Both measures were significantly different between neuron subtypes (Student’s t-test, *p* = 1.33e−06, and *p* = 6.79e−09, respectively). Baseline firing rates of bursting neurons were significantly faster (10.66 ± 7.20 Hz; Student’s t-test, *p* = 0.02) than those of non-bursting neurons (7.05 ± 4.51 Hz).

We also classified neurons based on their waveform width (i.e. trough-to-peak duration of the spike waveform; Fig. 5c), as spike waveform asymmetry measures have been reported to be one of the best predictors of neuron classification into functional groups^29^: interneurons (i.e. narrow waveform neurons) and pyramidal (i.e. wide waveform) neurons. We found a bimodal distribution of waveform widths (Fig. 5c), from which neurons were classified as either narrow waveform neurons (i.e. putative interneurons; n = 6, trough-to-peak width: 211.11 ± 17.21 µs; Fig. 5d) or wide waveform neurons (i.e. putative pyramidal neurons; Fig. 5d; n = 51, trough-to-peak width: 526.80 ± 72.12 µs). As expected, putative pyramidal neurons (n = 51; 89.5%) outnumbered putative interneurons (n = 6; 10.5%). Putative interneurons were all characterized by a Mode 2 spike-train autocorrelation histogram and were all classified in the non-bursting cluster. On the other hand, putative pyramidal neurons had heterogeneous modes of firing (Modes 1, 3 and 4). Mode 1 neurons were all classified in the non-bursting cluster. We speculate that Mode 1 neurons were likely ‘regular-spiking’ neurons, term used in intracellular recording studies. Regular-spiking neurons have wide waveforms, are abundant in the cortex and do not typically produce bursts^41,43^. Modes 3 and 4 were classified as bursting neurons. Mode 3 was found in only 1 neuron out the whole dataset, while Mode 4 was the second most predominant mode of firing in the dataset after Mode 1. We speculate that the neuron with a Mode 3 autocorrelation histogram was likely a ‘chattering’ neuron, described in intracellular recording studies as a type of bursting neuron that typically produces repetitive bursts with high intra-burst frequency^41,43^. The Mode 3 neuron frequently produced bursts with 2 or 3 spikes per burst (Supplementary Figure S3), with high intra-burst frequency (i.e. 2–3 ms interspike interval) followed by periods of inactivity. Chattering neurons are pyramidal neuron subtypes and are mostly found in superficial layers (i.e. II/III), rather than in the deep layers (i.e. V/VI) of the cortex^43^. On the other hand, another type of bursting neuron described in intracellular recording studies as ‘intrinsic bursting’, produces bursts with lower intra-burst frequency at the beginning of a depolarization current pulse, followed by tonic discharges^41^. Intrinsic bursting neurons are typically identified morphologically as large pyramidal neurons in layer V of the cortex. We speculate that Mode 4 neurons were likely intrinsic bursting neurons.

All neurons were clustered in the expected cluster using the *k*-means algorithm, except for one Mode 4 neuron clustered as a non-bursting neuron. Even though Modes 2 and 4 had similar autocorrelation median values, the difference in activity in bins ≤ 5 ms was sufficient to cluster neurons correctly. These results are summarized in Table 1.

**Table 1:** Electrophysiological properties of the four modes of neuron firing identified from the spike-train autocorrelation histograms computed from the initial 350 spikes recorded during pre-BMI baseline.

Mode of neuron firing	Autocorrelation median (ms)	Probability of firing in ≤ 5 ms (%)	Waveform width (µs)	*k*-means cluster
Mode 1 (n = 26)	33.64 ± 4.24	1.25 ± 1.54	502.58 ± 82.11 (Wide)	Non-bursting
Mode 2 (n = 6)	28 ± 1.14	0.74 ± 0.45	211.11 ± 17.21 (Narrow)	Non-bursting
Mode 3 (n = 1)	11.9	27.62	600 (Wide)	Bursting
Mode 4 (n = 24)	27.58 ± 1.78	6.47 ± 2.49	559.09 ± 58.07 (Wide)	Bursting

Data represents mean ± s.d

We also evaluated the performance of the clustering method using the probability of firing in ≤ 10 ms. In that case, three Mode 2 neurons and one Mode 1 neuron were classified as bursting neurons. We decided using the probability of firing in ≤ 5 ms as more suitable, as it did not split Mode 2 neurons into different clusters, and also based on the criteria described above.

### Bursting and non-bursting neurons improved reward frequency but only bursting neurons increased the magnitude of the event integrals

Finally, we investigated the change in average firing rate, event (i.e. rewarded and unrewarded) frequency and event integral during the up-regulation protocol, for DNs with significant up-regulation (i.e. learners) classified as bursting and non-bursting neurons.

**Figure 6:**
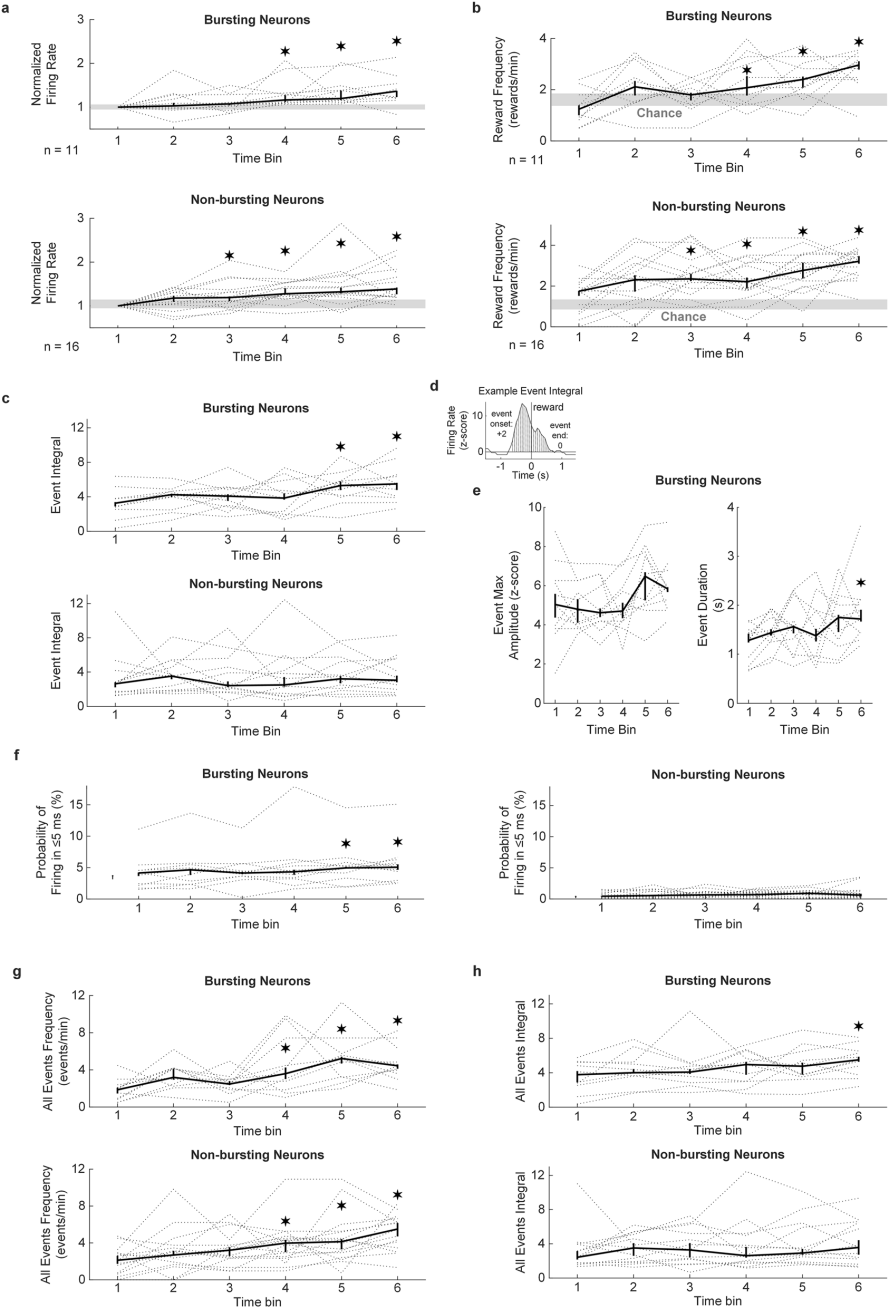
Differences in the response of bursting and non-bursting neurons during operant conditioning. (**a**) Firing rate per neuron subtype during the up-regulation protocol, for each of 6 equally-sized time bins. The firing rate was normalized to the firing rate during the first bin of the up-regulation protocol. The gray area denotes the 40th and 60th percentile range of the firing rate during pre-BMI baseline, normalized to the firing rate during the first bin of the up-regulation protocol. (**b**) Reward frequency per neuron subtype during the up-regulation protocol, for each equally-sized time bin. The gray area denotes the 40th and 60th percentile range of the chance performance during pre-BMI baseline. **c**. Rewarded event integral per neuron subtype during the up-regulation protocol, for each of 6 equally-sized time bins. (**d**) Example event aligned to reward (0 s) from which an event integral was calculated from event onset to end. (**e**) Rewarded event maximal amplitude and duration for bursting neurons. (**f**) Probability of firing in ≤ 5 ms per neuron subtype. These values were estimated for each time bin by computing the spike-train autocorrelation and normalizing by the number of spikes in each time bin. (**g**) All events (i.e. rewarded and unrewarded) frequency per neuron subtype. (**h**) All events (i.e. rewarded and unrewarded) integral per neuron subtype. For all plots: gray dotted lines indicate individual neurons. Bold line is the median with 40th and 60th percentiles (*p < 0.01, Wilcoxon’s signed-rank test, Bonferroni corrected).

First, we found that both bursting (n = 11) and non-bursting (n = 16) neurons underwent similar increases in firing rate and reward frequency, compared to the first bin of the up-regulation protocol, starting at time bin 4 (6.36 ± 1.21 min) and 3 (4.55 ± 1.60 min), respectively (Wilcoxon’s signed-rank test, Bonferroni corrected, *p* < 0.01; Fig. 6a,b). While non-bursting neurons underwent these learning-related changes slightly faster than bursting neurons, the peak reward frequency was not different at the end of the up-regulation protocol (bin 6, Wilcoxon’s rank-sum test, *p* = 0.17). In addition, chance performance during pre-BMI baseline was not different to the reward frequency during the first bin of the up-regulation protocol, for bursting (Wilcoxon’s signed-rank test, *p* = 0.46) and non-bursting (*p* = 0.36) neurons. Chance performance (Wilcoxon’s rank-sum test, *p* = 0.36), as well as the reward frequency during the first bin of the up-regulation protocol (*p* = 0.67) was not different between neuron subtypes.

Next, we found that the rewarded event integral was significantly larger during the last two bins of the up-regulation protocol, for bursting neurons (Fig. 6c; Wilcoxon’s signed-rank test, Bonferroni corrected, *p* < 0.01), while no difference was observed for non-bursting neurons. Then, we investigated if the elevated rewarded event integral in bursting neurons was the result of larger firing rates or prolonged event durations. An example event integral is shown in Fig. 6d. We found that the rewarded event duration was significantly longer during the last bin of the up-regulation protocol (Fig. 6e; Wilcoxon’s signed-rank test, Bonferroni corrected, *p* < 0.01). The maximal amplitude recorded for each rewarded event was not different throughout the up-regulation protocol. However, the probability of firing in ≤ 5 ms significantly increased for bursting neurons during the last 2 bins of the up-regulation protocol (Fig. 6f; Wilcoxon’s signed-rank test, Bonferroni corrected, *p* < 0.01), while no change was found for non-bursting neurons, suggesting that higher bursting activity might also be related to the increase in the rewarded event integral found in bursting neurons. Otherwise, this change might only indicate that as bursting neurons become more active, they produce more bursting events.

Finally, we investigated if there was a difference between bursting and non-bursting neurons in terms of all events (i.e. rewarded and unrewarded) frequencies and integrals. We found that both neuron subtypes significantly produced more events as the protocol progressed, starting in bin 3 (Fig. 6g; Wilcoxon’s signed-rank test, Bonferroni corrected, *p* < 0.01). However, only bursting neurons produced significantly larger all-event integrals during the last bin of the up-regulation protocol (Fig. 6h; Wilcoxon’s signed-rank test, Bonferroni corrected, *p* < 0.01), while non-bursting neurons did not.

## Discussion

We implemented single neuron operant conditioning of firing rates in a rat model and found successful activity up-regulation in 27 out of 57 neurons. Successful conditioning appeared to be the result of learning, as rats showed greater ability to produce up-regulation of firing rates as training progressed from days 1–3 compared to days 10–13. On average, direct neurons (DN) had gradual increases in firing rate, improved reward frequency, robust modulation around the reward contingency and increased all-event frequency as the up-regulation protocol progressed. In DNs with significant up-regulation, bursting neurons had additional learning-related changes in the increased activation (i.e. larger rewarded and unrewarded event integrals) towards the end of the up-regulation protocol and elevated bursting activity. The presence of these indicators was neuron-type-specific, which might have implications in the design of BMIs.

A similar study^12^ also found learning-related changes in the event rate and amplitude of conditioned neurons. In our study, high but short bursts of activity would not result in reward, thus learning-related changes had to involve sustained high firing rates (i.e. at least for 750 ms) to produce an event that would result in a reward, which only bursting neurons improved at, beyond the reward contingency. We also found that longer event duration, not maximal amplitude, was the likely mechanism behind larger event integrals in bursting neurons. This was probably a reflection on the reward contingency, which required sustained high firing rates. A proposed mechanism underlying the changes observed in bursting neurons is intrinsic plasticity. Learning, as well as some forms of experience-dependent plasticity, elicit intrinsic plasticity, which is manifested as an increase in the rate of firing of a neuron (for review^44,45^). Changes in intrinsic plasticity are expressed as changes in neuronal excitability and can be long-lasting (i.e. up to many days^46^). Classical conditioning alters intrinsic excitability^46,47^, as well as other forms of learning, including spatial learning^48^, fear conditioning^49^, odor discrimination^50^, and experiencing new environments^51^.

Long-term potentiation of intrinsic excitability (LTP-IE) has been induced in intrinsic bursting (i.e. identified morphologically as thick-tufted neurons projecting subcortically) layer V neurons in visual cortex after periods of repetitive high frequency stimulation in bursts^52^. High-frequency stimulation delivered in bursts has also been used to induce intrinsic excitability of deep cerebellar nuclear neurons^53^. Recent studies have shown that intrinsic excitability is altered only in neurons directly active during learning^54^, although, it has been shown to have global effects as well (for review^44^). Many forms of plasticity are layer- and neuron-type-specific^55–58^. Also, intrinsic plasticity shares common learning rules with synaptic plasticity, for example, with Hebbian- and spike-timing-dependent plasticity (for review^59,60^). Regarding our findings, neuron-type-dependent plasticity might also play a role in explaining the lack of changes in non-bursting neurons’ event integral.

A previous study found a dichotomy of experience-dependent plasticity in the two major types of neurons found in layers Va and Vb: regular-spiking and intrinsic bursting^61^, which are both pyramidal neuron subtypes. Even though their study was performed in the barrel cortex, the same principles might be generalized to layers Va and Vb of the motor cortex. The authors found that these neuron subtypes experience complimentary forms of synaptic plasticity in response to whisker trimming, where intrinsic bursting neurons were potentiated in the spared whisker barrels, while regular-spiking neurons were depressed in the trimmed barrels. Extrapolating their results, the bursting neurons (i.e. Mode 4/pyramidal) found in our study are likely candidates for intrinsic bursting neurons, while non-bursting neurons (i.e. Mode 1/pyramidal) are the most likely candidates for regular-spiking neurons. Similarly, we found bursting neurons to be potentiated (i.e. producing larger event integrals), while non-bursting neurons were neither potentiated nor depressed, possibly due to the nature of the BMI task involving activity up-regulation. Different BMI tasks, or perhaps extinction of existing BMI mappings might involve depression in the activity of non-bursting neurons. There is also the possibility that plastic changes act in a faster time-frame in bursting compared to non-bursting neurons, for the simple fact that intrinsic bursting neurons fire at high rates or in bursts of activity. Elucidating the specific cellular and molecular mechanisms underlying the plastic changes observed in neurons as a result of operant conditioning, and in particular, if differences exist between bursting neurons and non-bursting neurons in layer V of the motor cortex will be of interest in future studies.

Another possible explanation to our findings might be that pyramidal neuron subtypes have different strategies to perform a BMI task. A recent study reported different strategies in subtypes of interneurons performing a calcium-based BMI task in mice^24^. The task involved the ensemble activity of a pair of interneurons. In two subtypes of interneurons (i.e. somatostatin- and vasoactive intestinal peptide-expressing interneurons), activity frequency (i.e. frequency of calcium events) of the increasing neuron grew larger while activity frequency of the decreasing neuron remained the same. When parvalbumin-expressing interneurons were targeted, activity frequency of the decreasing neuron diminished, while activity frequency of the increasing neuron remained the same. These findings highlight the differences in plasticity in another major type of cortical neuron: interneurons. Interestingly, the authors reported that the amplitude of the calcium events did not change significantly, only the frequency of the events of individual neurons according to their arbitrary mapping (i.e. increasing or decreasing). The overall reward frequency between neuron subtypes was not significantly different. Given that in our study, we recorded from layer V, where pyramidal neurons are abundant, the proportion of interneurons (i.e. narrow-waveform neurons) was very low (i.e. 6 out of 51). Out of these 6 putative interneurons, only 2 were successfully conditioned, thus we are unable to draw conclusions regarding differences in the response of interneuron subtypes. However, we also observed subtype-specific strategies in putative pyramidal neurons, reflected in the magnitude of the event integrals, which highlights the importance of investigating how neurons are modulated in various neuroprosthetic tasks and how extrinsic and intrinsic factors can inform the design of BMIs.

Regarding the performance of non-bursting neurons in the BMI, the firing rate and reward frequency increased slightly earlier in the protocol than that of bursting neurons. However, this difference might be due to the effect size (i.e. non-bursting neurons: n = 16 vs. bursting neurons: n = 11), indicating that non-bursting neurons had increased power when performing statistical comparisons. Furthermore, it could be argued that producing larger events was not necessary, since the reward contingency remained the same throughout the experiment. In this case, non-bursting neurons produced a more stable response (i.e. stable event integral) leading to a reward than bursting neurons, whose response went beyond the reward contingency (i.e. overshoot).

Our findings in DNs with significant up-regulation (i.e. learners) suggest that control of the BMI was goal-directed. Successful activity up-regulation in DNs was not explained by delivery of rewards without LED feedback (i.e. rewards-only protocol), or a positive association with a flashing light without rewards (i.e. LED-only protocol), as both conditions had significantly lower average firing rates compared to the last 3 min of the up-regulation protocol. This was true even during the 1-SD BMI protocol, where significantly lower firing rates were produced compared to the second half of the up-regulation protocol, despite rewards being delivered more frequently and having higher LED brightness due to a lower reward threshold. This result suggests that changes in firing rate were the result of learning, as the average neuron activity only increased when a challenging reward threshold was presented, and an association was made between high firing rate and proportional high brightness of the LED. The presence of this association, however, was meaningless without food rewards (i.e. LED-only protocol), as the firing rate quickly returned to baseline levels, which indicates that the learned task was goal-directed rather than a learned habit. It should be taken into consideration that neurons were exposed to each modified protocol for 3 min at a time, which might have limited the amount of learning, especially during protocols involving rewards. One study exposed mice extensively in a single neuron operant conditioning task with only rewards^23^. However, the presence of sensory feedback seems to be necessary to produce a robust response around the reward contingency, selectively to DNs, as well as to expedite learning, as reported in this and other studies^10–12^. Also, DNs and INs were modulated at trial onset during the up-regulation protocol and this modulation was not present during LED-only, protocol in which rewards were not dispensed upon reaching the contingency, further suggesting volitional modulation of firing rates.

Other studies have reported the same specificity of single neuron responses in layers II/III and V in the cortex using operant conditioning. The mechanism by which single neurons can be volitionally modulated without simultaneous activation of neighboring neurons is unknown, however, it involves reward-modulated spike-timing-dependent plasticity^62,63^. Also, downstream circuits, namely the basal ganglia-thalamo-cortical loop, regulate plasticity in dopaminergic neurons that project to the motor cortex (for review^64^). Corticostriatal plasticity is reported to play a crucial role in BMI learning^10^. However, one study found evidence that upstream circuits play a role as well^12^. They tested a condition in which mice had to initiate licks upon trial completion in order to get a liquid reward, therefore mice would have to rely on internal cues to maintain performance after conditioning. They found that mice were not able to predict reward outcomes based on internal cues alone, therefore sensory cues remained necessary even after conditioning. This evidence would suggest that motor cortex neurons are driven by downstream reward-related circuits, and upstream circuits are then able to hone in to an individual output neuron. In our study, further evidence of this would be the fact that both DNs and INs were modulated at trial onset, however, modulation and performance improvement at the time of reward were confined to DNs, indicating that INs likely played a role in providing part of the drive to DNs during conditioning, as reported in several BMI studies^12,20,22,25,26^. Although, IN modulation has been reported to decrease with prolonged learning^22,25^. One study reported that both DNs and INs experienced increased phase-locking and coherency to slow-wave activity during sleep, suggesting a supportive role for INs in the consolidation of BMI skills^26^.

Despite the marked learning-related changes found in almost half of the neurons, the other half did not up-regulate their activity as a result of operant conditioning (i.e. 30 out of 57), regardless of neuron subtype. One study trained mice to pull a lever prior to operant conditioning of single M1 neurons^23^. They reported that only neurons which were not modulated to the lever-pull task were successfully conditioned. Even though we did not test this condition, an association to existing motor commands might explain why some neurons, regardless of neuron subtype, did not respond to operant conditioning.

In the context of BMIs implementing neural population decoding for real-time control of artificial limbs or computer cursors, one study investigated neuron-type-specific utility, defined as improved offline decoding of motor parameters, in narrow and wide waveform neurons^27^. The authors reported that narrow waveform neurons outperformed wide waveform neurons in various motor parameters. It remains to be investigated how the different responses in cortical neuron subtypes, and subtypes, impacts the decoding accuracy of dynamic signals, such as torque, EMG or force. The progressively larger response at the time of reward found in bursting neurons might lead to higher utility in BMI implementation, as it will likely carry more information for a given decoder to translate into commands related to motor execution or intention, ultimately requiring less neurons to achieve the same level of performance as a larger number of neurons with lower utility (i.e. non-bursting neurons).

## Conclusions

When designing a BMI system, one should consider the different neuron-type-specific responses when learning a novel BMI task. Wide waveform (i.e. putative pyramidal) neurons are an abundant source of output signals in the cortex, capable of producing robust responses within minutes as a result of operant conditioning. However, bursting neurons produced larger event integrals at the time of reward, which were a result of sustaining high firing rates for longer than non-bursting neurons. These additional learning-related changes might be indicators of higher utility observed in bursting neurons. With respect to neuron classification, computing the spike-train autocorrelation will identify the mode of firing, revealing the bursting propensity, or lack of it, of individual neurons.

## Limitations of the study

Our study is limited by the fact that rats were unrestrained during experiments. We did not determine whether the direct neurons had a previous association to forepaw limb movements, nor did we track movements or EMG activity. However, we did not observe overt behavioral strategies that could be up-regulating the single neuron activity. In addition, activity of DNs was selectively modulated around reward dispensing, unlike activity of INs. If rats relied on stereotyped forepaw moments to obtain rewards, the local circuit would have been driven in concert. Finally, the modified protocols were aimed at determining whether the increase in firing rate and reward frequency during the up-regulation protocol was volitional. Nonetheless, inclusion of movement tracking or intramuscular EMG in future experiments will allow us to ascertain that control of the BMI was volitional.

## Methods

### Animal model and experimental setup

Nine adult male Long-Evans rats underwent stereotaxic surgery for electrode implantation in the forepaw representation of the primary motor cortex (M1), targeting layer V at a depth of ~ 1,500 µm. The University of Toronto Animal Care Committee approved the protocols described below. All experiments were performed in accordance with relevant guidelines and regulations. The electrode consisted of an array of sixteen Parylene C-insulated tungsten electrodes (Microprobes for Life Sciences, Gaithersburg, MD, USA; 4 × 4 configuration; 250 μm inter-electrode distance; impedance of ~ 0.5 MΩ at 1 kHz). Rats recovered from surgery for 10 days before taking part in the experiments. Analgesia (i.e. Meloxicam at a dose of 1–2 mg/kg administered subcutaneously) was provided once per day during the first 2–3 days post-surgery.

Operant conditioning experiments were performed in a chamber (Med-Associates Inc, St. Albans, VT, USA) equipped with a food dispenser that dispensed 45 mg chocolate-flavored pellets (Bio-Serv, Flemington, NJ, USA), and a light-emitting-diode (LED) for visual feedback. A buzzer was added to the chamber to indicate trial onset during the experimental protocols. The chamber components (i.e. pellet dispenser, LED, and buzzer) were controlled in real-time with custom-made routines created using MATLAB (Mathworks, Natick, MA, USA) and a microcontroller board (Arduino UNO, Ivrea, Italy).

### Single neuron recordings

Neural activity was recorded at 30 kHz, band-pass filtered from 250–5,000 Hz and digitized with 16-bit resolution using an amplifier and data acquisition system (Cerebus, Blackrock Microsystems, Salt Lake City, UT, USA). Spikes were sorted using time–amplitude windows, which were placed manually on the spike waveform^65^. The criteria used to select a neuron for subsequent experimentation included: a minimum refractory period of 1 ms^66^, a signal-to-noise ratio (SNR) of two or greater, as described in^67^, minimum firing rate > 1 Hz and a unit not conditioned previously. Refractory period violations were assessed using an interspike interval (ISI) histogram from the pre-BMI baseline recordings (described below). SNR was calculated as the ratio between the peak-to-peak amplitude of the mean neuron waveform, divided over two times the s.d. of the waveform residuals after the mean waveform was subtracted.

### BMI task and experimental protocols

Once a neuron was selected, its average firing rate and ISI histogram were computed from a 5-min baseline recording (i.e. pre-BMI baseline). Rats were tethered to the headstage but otherwise unrestrained during the entire experimental timeline (Fig. 3e). During this recording, the rats received no auditory cues, visual feedback or food rewards. The recorded activity was used to determine the firing rate distribution of the selected neuron, from which the high activity reward threshold (i.e. 2 s.d. above baseline firing rate) was determined for subsequent protocols.

During the “up-regulation” protocol (i.e. the protocol in which the BMI task was performed), the neuron’s firing rate was transformed into the brightness of the LED for visual feedback (Fig. 1a). The purpose of the up-regulation protocol was to induce a gradual increase of the neuron’s firing rate, where at the beginning, rewards were obtained infrequently, however, as the overall firing rate of the neuron increased, rewards would become more frequent. The presence of up-regulation (i.e. significant firing rate increase from the first to the second half of the protocol) was our indicator for successful conditioning. To provide LED visual feedback, the brightness of the LED was adjusted automatically in linear increments using pulse-width modulation to reflect the firing rate of the neuron (Fig. 1b). The LED displayed maximum brightness when the firing rate reached the high activity threshold and the minimum brightness corresponded to no activity. Firing rates were updated every 250 ms. A trial started with an audio cue (i.e. 750 ms ‘beep’ produced by the buzzer) and the simultaneous activation of the LED. The reward contingency consisted of reaching the high activity reward threshold and sustaining it for at least 750 ms (i.e. 3 consecutive bins). Upon reaching it, a pellet was immediately dispensed, the LED was turned off and the rat was given 10 s to retrieve the pellet. A new trial started when the firing rate went back to baseline activity levels. There was no time limit to complete a given trial. The up-regulation protocol consisted of a total of 20 trials (Fig. 1c).

After the up-regulation protocol, a second baseline (i.e. post-BMI baseline) was recorded for a minimum of 5 min, or until the average firing rate of the conditioned neuron went back to pre-BMI baseline levels.

Two of three modified (i.e. control) protocols were performed in addition to the up-regulation protocol during a given conditioning session. These three modified protocols involved a reward threshold of 1 s.d. above pre-BMI baseline firing rate and were conducted to test the effect of: (i) LED-only (i.e. no rewards; Fig. 3a); (ii) rewards-only (i.e. no LED feedback; Fig. 3b); (iii) LED feedback and rewards with a lower threshold (i.e. 1-SD BMI). The modified protocols were chosen as follows: since two of them involved rewards (i.e. rewards-only and 1-SD BMI), one of these protocols was chosen at random; the LED-only protocol was always performed. One modified protocol was conducted after pre-BMI baseline and before the up-regulation protocol; the second modified protocol was conducted after post-BMI baseline (Figs. 3c,d). The order in which the modified protocols were performed was randomized as well. Trial onset was indicated with a beep as in the up-regulation protocol. The modified protocols lasted 3 min each. The experimental timeline is shown in Fig. 3e.

Rats underwent conditioning for as many days as we were able to obtain good quality single neuron recordings (i.e. SNR > 2). Only conditioning sessions in which 20 trials were completed during the up-regulation protocol were included in subsequent analyses. One conditioning session was performed per day, per animal. Neurons with baseline firing rates < 1 Hz were discarded from the analyses because we established that these neurons lack utility in a BMI in a previous study^28^.

### Neuron classification

Neurons were classified post-hoc into bursting and non-bursting neurons, based on two measures of their spike-train autocorrelation histograms. The spike-train autocorrelation quantifies the probability of spikes being generated at a given time interval, therefore, it can characterize the firing behavior of a neuron. Pre-BMI baseline recordings were used to compute the spike-train autocorrelation at 1 ms resolution, from − 50 to 50 ms. Recordings were truncated at 350 spikes. The center bin (i.e. 0 ms lag) was discarded from the autocorrelation histograms. In order to classify neurons based on their autocorrelation histograms, we first calculated the autocorrelation median^34^, which consisted of the positive time lag where half the histogram weights occurred. The autocorrelation median was computed at 0.1 ms resolution. Second, the probability of firing in ≤ 5 ms, calculated as the percentage of histogram weights in ≤ 5 ms divided over the total autocorrelation histogram weights.

These two measures were chosen based on visual inspection of the main modes of firing shown in Fig. 5a, identified from the neuron autocorrelation histograms. Non-bursting neurons (i.e. Modes 1 and 2; Fig. 5a) were characterized by very low firing activity in bins ≤ 5 ms (i.e. < 2% of the autocorrelation histogram bin count). Mode 1 neurons were identified by a slow rise in the histogram from 0 to 50 ms, resulting in autocorrelation medians > 30 ms. Mode 2 neurons also had very low activity in bins ≤ 5 ms, however, these neurons had a peak in their autocorrelation histograms between 15–30 ms, resulting in autocorrelation medians between 27–30 ms. Bursting neurons (i.e. Modes 3 and 4; Fig. 5a) described neurons with higher activity in bins ≤ 5 ms. Mode 3 illustrates the bursting neuron described in the literature^29^, characterized by a peak in the autocorrelation histogram between 3–6 ms, followed by exponential decay, resulting in autocorrelation medians < 15 ms. Mode 4 neurons had a very wide firing range, including bins ≤ 5 ms. Even though Mode 4 neurons did not solely produce bursting activity, they were numerous in the recordings and were distinguished from non-bursting Mode 2 neurons on the basis of their higher probabilities of firing between 2–5 ms. For visual inspection, autocorrelation histograms were constructed using all available spikes from each neuron (Fig. 5a). However, for classification purposes, only spikes recorded during pre-BMI baseline (i.e. spontaneous activity) were considered. During the up-regulation protocol, some neurons increased their activity as a result of conditioning. However, other neurons decreased their activity (see Mode 3 neuron in Supplementary Figure S3). Due to this variability during the up-regulation protocol and given that our classification system was activity-based, we considered that classifying neurons based on their spontaneous activity would be more consistent. Also, the same number of spikes per neuron (i.e. the first 350 spikes) during pre-BMI baseline were used to compute the autocorrelation-based measures to facilitate between-unit comparisons^68–70^. Finally, we used *k*-means clustering^71,72^ to classify neurons into non-bursting and bursting neurons from the autocorrelation median and probability of firing in ≤ 5 ms.

We also calculated the trough-to-peak width of the average neuron waveform shape during pre-BMI baseline (Fig. 5d), given that spike waveform asymmetry measures have been reported to be the best predictors of neuron classification into functional groups (i.e. interneurons and pyramidal neurons^29^. Based on this definition, putative interneurons are characterized by narrow or ‘thin’ spikes and they are quite scarce in the cortex (i.e. < 30%, although numbers as low as 7% have been reported^29,30,41^). In contrast, putative pyramidal neurons have wide spikes and are very numerous in the cortex (i.e. 60–70%).

### Data analysis

Statistical analyses were performed in MATLAB (2018a, Mathworks, MA, USA). Kolmogorov–Smirnov tests were used to determine if the data met the normality assumption. Non-parametric tests were used where appropriate. All tests were two-sided unless otherwise stated. All tests were performed with a significance level (α) of 0.05. Data are presented as mean ± s.d.

Firing rates were quantified in bins of 250 ms. Neurons with significant increases in firing rate (Wilcoxon’s rank-sum test, *p* < 0.05) from the first to the second half of the up-regulation protocol were considered successfully conditioned.

Conditioning sessions with paired modified (i.e. control) protocols were compared to the last 3 min of the up-regulation protocol (Wilcoxon’s signed-rank test, Bonferroni corrected, *p* < 0.017).

For each successfully conditioned neuron, we divided the up-regulation protocol into 6 equally-sized time bins, from which we constructed learning curves per neuron subtype of firing rate, reward frequency and other measures described below. This allowed for paired statistical comparisons. Firing rates and chance performance during pre- and post-BMI baseline were calculated for equivalent time bins, as in the up-regulation protocol. Firing rates were normalized to the firing rate during the first bin of the up-regulation protocol.

The learning effect (Fig. 2a) was estimated by taking the average firing rate change factor for each consecutive day of training in all rats. The firing rate change factor was computed by taking the mean firing rate during the second half of the up-regulation protocol, divided by the mean firing rate during the first half of the protocol. Data was pooled for training days 1–3, 4–6, 7–9 and 10–13.

We assessed the rewarded and unrewarded events (i.e. firing rate evens that reached or surpassed the reward contingency) by transforming the firing rates to a *z*-score (Fig. 6d), where the spikes were first binned every 62.5 ms, for greater resolution of the firing rates, and then smoothed with a sliding window of 250 ms, consistent with the bin width during experimentation. We calculated the mean and s.d. during the first 25 s of the up-regulation protocol to transform the firing rate histograms to a *z*-score waveform. This time window was chosen because rewards were infrequent at the beginning of the up-regulation protocol, and thus, firing rates were not yet elevated. Events were detected when the *z*-score waveform first crossed a value of 2 (*p* < 0.023), and until the waveform returned to a value below 0. From these two time points, the event duration and amplitude were used to calculate an event integral (i.e. sum of *z*-score transformed firing rates from event onset to end).

To examine co-activation of other neurons recorded in the array along with the conditioned neuron, a peri-event time histogram (PETH) was constructed using 5 s around and after the reward contingency for each neuron, from − 3 s to + 2 s, with a bin size of 62.5 ms to assess firing rates in a finer timescale (Fig. 4a). Neurons in the array with average firing rates of < 1 Hz were not included in the analyses. The raw PETH was transformed to a *z*-score waveform using the mean and s.d. of the PETH from − 3 to − 1 s. Co-activation in neighboring neurons was detected when the *z*-score PETH trace crossed a value of 2 (*p* < 0.023) for at least 4 consecutive bins (i.e. 250 ms, or the bin width during conditioning). These neurons were labeled indirect neurons (IN), while conditioned neurons were labelled direct neurons (DN). The modulation depth was calculated for DNs and INs around the reward contingency, from − 1 to 0.5 s, and after reward contingency, from 0.5 to 2 s. The modulation depth was calculated as the difference between the maximum and the minimum *z*-score firing rate values within each respective window. Similar to the reward-aligned PETH, we constructed a trial onset-aligned PETH, from − 0.5 to + 1 s, with a bin size of 62.5 ms, for DNs and INs. The trial onset-aligned PETH was transformed to a *z*-score trace by taking the mean and s.d. from − 0.5 to 0 s. The modulation depth was calculated as the difference between the maximum and minimum *z*-score firing rate value within this window.

## Supplementary information

Supplementary Information.

## Data Availability

The raw datasets generated during the current study are available from the corresponding author on reasonable request. All data analyzed during this study are included in this published article and supplementary materials.
