# Supplementary material for: Operant conditioning of motor cortex neurons reveals neuron-subtype-specific responses in a brain-machine interface task
Source: Sci Rep. 2020 Nov 17;10:19992. doi: 10.1038/s41598-020-77090-2 (PMC7672061; doi:10.1038/s41598-020-77090-2)
Supplement: Supplementary file 1 — Supplementary Information. [file 41598_2020_77090_MOESM1_ESM.docx]

Title: Operant conditioning of motor cortex neurons reveals neuron-subtype-specific responses in a brain-machine interface task

Authors: Martha Gabriela Garcia-Garcia, Cesar Marquez-Chin, and Milos R. Popovic





Figure S1. Example neuron waveforms. Eight different single units are shown, with 150 spikes each.





Figure S2. Time to reward contingency for learners. Proportion of the time to reach the reward contingency for neurons labelled as learners, for the first and second half of the up-regulation protocol.





Figure S3. Example raster plots from each mode of firing. Raster plots showing the 4 modes of firing as seen in the raster plots for 3 different timepoint during recording. The arrows in Mode 3 neuron indicate bursts of 2-3 spikes.
